# Experimental and Numerical Investigation into the Mechanical Behavior of Composite Solid Propellants Subject to Uniaxial Tension

**DOI:** 10.3390/ma16206695

**Published:** 2023-10-15

**Authors:** Chengfeng Wu, Ming Jiang, Yingying Lu, Hongjian Qu, Hongyan Li, Shaoqing Hu

**Affiliations:** Xi’an Modern Chemistry Research Institute, Xi’an 710065, China

**Keywords:** composite solid propellant, microstructural model, mechanical behavior, cohesive model, dewetting

## Abstract

To further explore the quasi-static mechanical characteristics of composite solid propellants at low strain rates, an investigation was conducted on the mechanical behavior and damage mechanisms of a four-component hydroxy-terminated polybutadiene (HTPB) propellant by means of experiments and numerical simulation. A uniaxial tensile test and scanning electron microscope (SEM) characterization experiment were carried out. A microstructural model, which accurately represents the mesoscopic structure, was developed via the integration of micro-CT scanning and image-processing techniques. The constructed microstructural model was utilized to conduct a numerical simulation of the mechanical behavior. The experimental results demonstrated that the maximum tensile strength increases with increasing strain rate, and the primary cause of propellant failure at low strain rates is the dewetting phenomenon occurring at the interface between the larger particles and the matrix. The maximum tensile strength is 0.48 MPa when the strain rate is 0.00119 s^−1^, and the maximum tensile strength is 0.37 MPa when the strain rate is 0.000119 s^−1^. The simulation results indicated a consistent trend in variation when comparing the simulation and experimental curves. This suggested that the established model exhibits a high level of reliability, and provides a promising approach for carrying out microstructural simulations of heterogeneous propellants in future. The mechanical behavior of the propellant can be effectively described by utilizing a mesoscopic finite element model that incorporates the superelastic constitutive model of the matrix and the bilinear cohesive model. This framework facilitates the representation of mesoscopic damage evolution, which consequently provides insights into the damage mechanism. Additionally, the utilization of such models assists in compensating for the limitations of damage evolution characterization experiments.

## 1. Introduction

A composite solid propellant is a type of energetic material that is formulated by combining various components such as a binder, an oxidizer, and metal particles in specific proportions. This material exhibits isotropic homogeneity on a macroscopic scale (ranging from millimeters to centimeters). However, on a mesoscopic scale (ranging from micrometers to millimeters), it displays a heterogeneous structure wherein solid particles act as the dispersed phase, and the binder acts as the continuous phase. Notably, distinct interfaces exist between these phases. The determination of the complexity of the mechanical properties of composite solid propellants is attributed to their specific compositional features. Numerous investigations have been undertaken to study the mechanical properties of solid propellants using continuum mechanics theory at the macroscopic level. Nevertheless, these studies have encountered difficulties in accurately capturing the internal phenomena of dewetting, damage, and failure processes that occur within propellants when subjected to external loads [1]. In recent years, a body of literature has focused on the mesoscopic structure of composite solid propellants. These propellants are regarded as multiphase composite materials, consisting of a binder matrix, solid particles, and the interface between the two. Researchers have constructed a mesoscopic mechanical model to accurately predict the macroscopic mechanical properties and failure modes of these composite solid propellants [2,3,4,5]. Hence, the investigation of the mechanical characteristics of composite solid propellants at the mesoscopic scale holds significant scientific significance.

To establish a mesoscopic mechanical model, it is necessary to construct a suitable RVE, i.e., a microstructural model. Mesoscopic mechanics is a two-scale mechanical structure including both the macroscopic scale and the mesoscopic scale [6]. At the macroscopic scale, the continuum consists of many material points, and the mesoscopic space associated with the macroscopic point is called the representative volume element (RVE), which is a fundamental concept in mesoscopic mechanics, and needs to satisfy the duality of scales. On the one hand, the size of RVE is small enough to be regarded as a material point at the macroscopic scale; thus, the macroscopic stress and strain fields in the RVE can be considered uniform. On the other hand, the size of RVE is large enough to contain many mesoscopic elements and sufficient mesoscopic structure information at the mesoscopic scale, so it can represent the statistical average properties of a localized continuum [7]. Thus far, there have been a lot of studies on RVEs for composite materials [8,9,10,11,12]. For instance, Zhang et al. [8] presented a parameterized and automated modelling method for generating 3D orthogonal woven composite RVE geometry, including yarn geometry variations. In this method, an RVE was obtained by geometrically transforming several basic yarns containing some geometrical features. Manuel et al. [9] presented an improved version of discrete RVE automation and framework generation. Several complex microstructure features, extracted from real microstructures, have been added to the generator to enable it to generate RVEs with realistic microstructures. Sun et al. [10] proposed a novel computationally efficient reconstruction of the 3D microstructure of an RVE model, with a real distribution of particles based on FIB-SEM techniques.

In addition, after completing the RVE modeling, it is important to establish the cohesive zone model. An interfacial layer with a certain adsorption energy is formed between the propellant binder and the solid particles, and the mechanical behavior of the interfacial layer will directly determine the macroscopic mechanical properties of the propellant. Therefore, to understand this physical mechanism, a traction–separation law needs to be introduced to describe the mechanical interaction between the matrix and the particles. The cohesive zone model can intuitively and accurately describe the bonding properties of the interface, and has been gradually applied to the simulation of bonded interfaces, which can define the crack initiation and propagation laws in the fracture process zone of the material. Currently, cohesive zone models proposed by scholars have various forms, among which the bilinear model [13] and exponential model [14] are typically used. Moreover, with the deepening of study on the fracture of different composites, a variety of atypical cohesive zone models have been established [15,16,17,18]. For example, Ghabezi [16] adopted a trapezoidal traction–separation law to investigate the mode II fracture in the presence of nanoparticles. Furthermore, Ghabezi [17] proposed a new piecewise function traction–separation law to simulate and predict fracture in mode I and mode II. However, the use of atypical cohesive zone model for numerical simulations in Abaqus 2021 commercial software requires the development of complex subroutines, which is extremely inconvenient. As a result, typical cohesive zone models have been widely used in numerical simulations of fracturing and damaging composite materials.

A crucial aspect in the establishment of the microstructural model is efficient control of the morphology, dimensions, and spatial arrangement of the internal particles. However, the composite solid propellant has a significant quantity of internal particles that are scattered in a random manner with precise gradation, which presents a considerable obstacle to establishing the microstructural model for the propellant. Currently, microstructural modelling methodologies for composite solid propellants may be broadly classified into two categories: (1) the parametric random generation method [19,20,21,22,23,24,25,26], which involves the generation of randomly distributed particles within a specified computational domain based on the shape, gradation, and volume fraction of solid particles in the composite solid propellant; and (2) the image processing method [27,28,29], which entails obtaining the real mesoscopic structure of the composite solid propellant through techniques such as SEM or CT scanning. Subsequently, different feature regions are identified using a threshold segmentation method, leading to the generation of 2D or 3D microstructural models.

The classification of parametric random generation methods may be expanded to include sequential filling algorithms and parallel filling algorithms [30]. The sequential filling algorithm involves the placement of particles with a predetermined shape into a filling domain during the initial stage. Once the first particle reaches a specified equilibrium state, subsequent particles are added incrementally until the desired volume fraction is achieved. The approach that is deemed a more representative algorithm is the random sequential algorithm (RSA) developed by Widom [31]. Subsequently, Rintoul et al. [32] added more optimizations and enhancements to the algorithm, which have since gained significant traction in the field of the mesoscopic characterization of particle-filled composites. Parallel algorithms typically involve the utilization of the Monte Carlo algorithm and molecular dynamics method to introduce a significant quantity of particles into the designated domain at the initial time step. The particles are subsequently rearranged and adjusted based on pre-established rules until the desired volume fraction is achieved. Stafford [33,34] proposed a random filling algorithm inspired by a molecular dynamics algorithm, which considers particles of diverse shapes. The algorithm determines collision times between particles by formulating a minimization problem within the framework of optimization theory. Stafford further demonstrated the efficacy of this approach by constructing a random filling system that accommodates various shapes, including cylinders, polyhedral, and spherical cylinders. Nevertheless, the integration of microscopic observation and image processing has garnered significant scholarly interest due to swift advancements in computer technology. For instance, Liu et al. [29] employed SEM to examine the surface of HTPB propellant, resulting in SEM images. These images were then subjected to binarization to extract features pertaining to particle edges. Ultimately, the particle filling model was constructed through the utilization of the least-squares fitting method.

Most mesostructure models generated with the parametric modeling approach consist of regularly shaped particles, such as spheres or circles. However, composite solid propellants contain many solid-phase particles, and most of these raw particles are irregularly shaped at mesoscopic scale. Therefore, the actual particles in the real mesostructure do not possess regular shapes. This discrepancy poses a challenge in accurately representing the meso-structure of composite solid propellants. The methodology of image-processing modelling involves the reconstruction of contours utilizing the original micro image. This approach enables the creation of a microstructural model that closely approximates the actual mesoscopic structure [35,36]. In brief, parametric modelling has been employed in plenty of publicly reported numerical simulation studies on the mechanical behavior of composite solid propellants based on microstructural models, whereas modeling based on the image recognition processing method has been reported infrequently. The present study aims to analyze the mechanical behavior and damage mechanism of a four-component propellant at low strain rates by means of experiments and numerical simulation. The microstructure of the composite solid propellant was modelled using image recognition processing, which involved analyzing micro-CT scanning images. Afterwards, the generated microstructural model was then converted into a vectorized format and imported into Abaqus 2021 finite element software to carry out numerical simulation. The accuracy of the numerical simulation results is assessed by a comparison with the corresponding experimental data. Meanwhile, a verification process was conducted to assess the applicability of employing this modelling method for investigating the mechanical characteristics of composite solid propellants.

## 2. Experimental Procedure

### 2.1. Material Preparation

**Figure 1:**
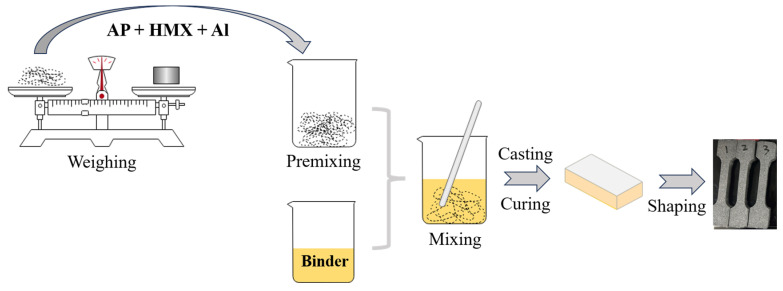
Preparation procedure of the composite solid propellant.

The composite solid propellant is a heterogeneous material that comprises solid particles as fillers and a binder as the matrix. The solid particles in this propellant have a size distribution ranging from several microns to hundreds of microns, resulting in a mesoscopic structure consisting of both the matrix and the solid particles. The composite solid propellant discussed in this paper is a four-component HTPB propellant with a solid particle content of 90 wt%, which mainly consists of non-energetic binder, non-energetic plasticizer, ammonium perchlorate (AP), cyclotetramethylene tetranitramine (HMX), aluminum powder (Al), etc. The material parameters and compositions are presented in Table 1. The propellant specimens were fabricated via a slurry casting process, in which a 25 L casting process line was adopted. The detailed procedure for preparing the specimens is illustrated in Figure 1.

### 2.2. Uniaxial Tensile Experiments

To investigate the mechanical response of the propellants under tensile loading, a series of uniaxial tensile experiments were conducted with different low strain rates at ambient temperature. In accordance with the prescribed guidelines outlined in the GJB 770B–2005 Method 413.1 of the People’s Republic of China, the tensile specimens were meticulously fabricated to a standard dumbbell shape with an effective size of 70 mm × 10 mm × 10 mm [37]. Furthermore, to eliminate the residual stresses inside the specimen, the specimen should be left for 24 h after processing before conducting the experiment. The uniaxial tensile experiments were carried out at three tensile rates, 0.5 mm/min, 2 mm/min, and 5 mm/min, and the corresponding strain rates were 0.000119 s^−1^, 0.000476 s^−1^ and 0.00119 s^−1^, respectively. Moreover, to ensure the reliability and generalizability of the experimental findings, it is imperative to obtain three sets of accurate data for each strain rate condition, which should then be averaged.

**Figure 2:**
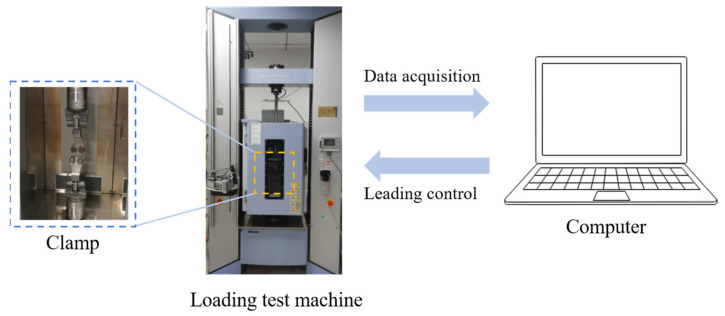
Diagram of the experimental apparatus.

Prior to conducting the experiment, the dimensions of the upper, middle, and lower portions within the central parallel region of the specimen were measured. The minimum value obtained from these measurements was selected as the initial cross-sectional area of the specimen. Following the completion of the measuring process, all specimens were subjected to a 12 h period at room temperature to maintain uniform internal and external temperatures for each specimen. The experimental machine is an electronic universal testing machine AG-X Plus 100, manufactured by the Shimadzu Corporation of Kyoto Japan, which has characteristics of ultra-high speed data acquisition with a 0.2 ms sampling interval, and can guarantee an accuracy of “±” 0.3% within the load sensor capacity range of 1/1 to 1/100. The test system consisted of a loading test machine and a computer, as shown in Figure 2.

### 2.3. SEM Characterization Experiments

In order to facilitate the analysis of the microscopic changes of the propellant under uniaxial tension, the micromorphology of the propellant before and after tension was observed via SEM. The fracture interface of the propellant specimen after the uniaxial tensile experiment was severed following the uniaxial tensile experiment. Subsequently, the obtained tiny fragments were affixed onto a metal block utilizing conductive glue, to undergo a surface gold-spraying treatment. This was followed by the examination and documentation of the specimen’s morphology via observation and photography. The experimental apparatus employed in this study was a QUANTA 600F field emission scanning electron microscope, manufactured by the FEI corporation of Hillsboro, OR, USA. During the observation process adopting SEM, the general morphology of the specimen was initially observed at a low magnification, followed by a more detailed observation of the specific local morphology at high magnification.

In addition, to clearly identify the particles in the CT image of the propellant, the raw microscopic shapes of AP and HMX powders were observed using SEM, which was performed in a similar procedure as above.

### 2.4. Micro-CT Scanning Experiments

To gain a comprehensive understanding of the distribution of each constituent of the propellant at the microscopic scale, a propellant square measuring 5 mm × 5 mm × 5 mm was subjected to micro-CT scanning without any external load. Micro-CT imaging operates on the principle of employing a micro-focus X-ray bulb tube to scan and transmit layers of the sample. The X-ray is received by a detector, which converts it into visible light and subsequently transforms it into an electrical signal with a photoelectronic converter. The electrical signal is then converted into a digital signal through an analog/digital converter, and ultimately processed by computer imaging. The experiment employed FF20 CT equipment, manufactured by the YXLON corporation of Hamburg, Germany. The apparatus was fitted with a 190 KV nano transmission tube, enabling a 2D detail resolution of up to 150 nm. Furthermore, the motion control mechanism included in the granite foundation guarantees temperature stability and achieves an exceptionally low thermal expansion, hence optimizing precision and accuracy.

## 3. Experiment Results and Discussion

### 3.1. Mechanical Response

**Figure 3:**
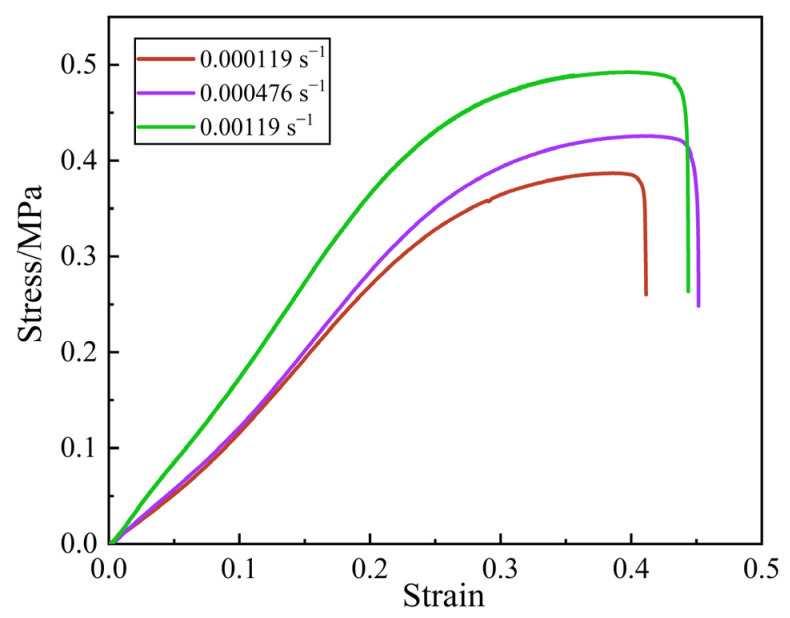
Stress–strain curves under different low strain rates at ambient temperature.

The stress–strain curves of the propellant under different strain rates at ambient temperature are depicted in Figure 3. Notably, strain is a relative value in Figure 3 Overall, the propellant exhibits typical nonlinear mechanical behavior. The mechanical properties of the propellant have a significant rate dependence. Furthermore, the greater the strain rate, the greater the maximum tensile strength of the propellant. When the strain rate is 0.00119 s^−1^, the maximum tensile strength is 0.48 MPa, and when the strain rate is 0.000119 s^−1^, the minimum tensile strength is 0.37 MPa.

When the magnitude of the strain is relatively low, the stress exhibits a linear relationship with the strain, indicating that the propellant possesses linear elasticity. This behavior is observed inside the linear region of the stress–strain curve. As the strain gradually grows, the propellant suffers internal damage, causing a minor deceleration in stress as the strain continues to rise. During this period, the material experiences a decrease in stiffness and exhibits a phenomenon known as softening. This softening behavior occurs in the nonlinear region of the stress–strain curve, which is located above the linear region. Once the applied strain beyond a specific critical threshold, the stress exhibits a discernible decline with the strain continues to grow. This is attributable to the fact that when the strain reaches the extreme value (about 0.45), the propellant particle dewetting phenomenon is significant, and the matrix is elongated to the point of tearing by completely bearing the external load, resulting in the macroscopic failure of the propellant within a short period of time [38]. This decline aligns with the decreasing segment of the stress–strain curve.

### 3.2. Micromorphology Analysis

**Figure 4:**
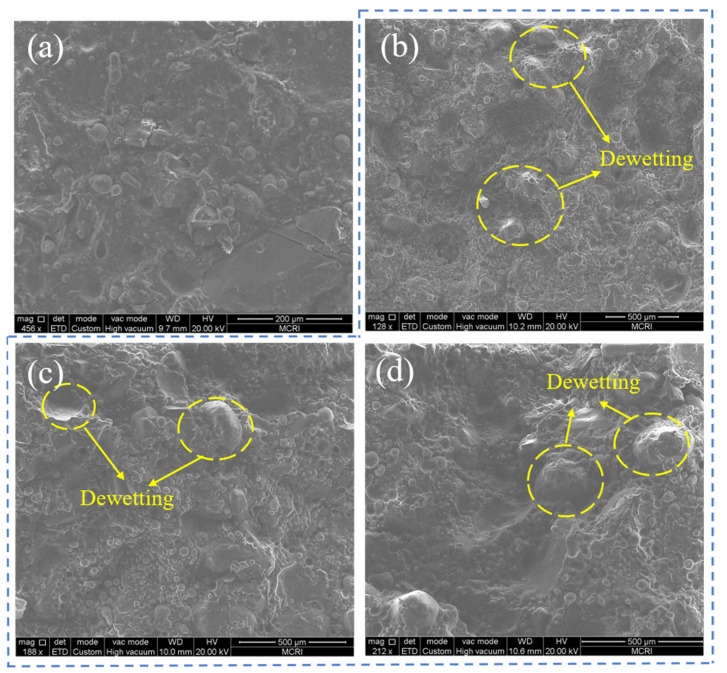
The micromorphology at different conditions: (**a**) before tension; (**b**) after tension at 0.000119 s^−1^; (**c**) after tension at 0.000476 s^−1^; (**d**) after tension at 0.00119 s^−1^.

The microscopic morphology of the propellant before and after tension is depicted in Figure 4. Comparing Figure 4b–d with Figure 4a revealed that the microscopic morphology of the propellant exhibits a relatively flat surface prior to stretching, whereas it exhibits obvious pits and bare particles after tensile fracture. Moreover, it is deduced that the damage mechanism of the propellant remains relatively consistent at low strain rates. This primarily manifests as the interfacial damage occurring between the larger particles (AP and HMX) and the matrix, commonly referred to as “dewetting”. Simultaneously, the phenomenon of dewetting between the smaller particles composed of Al and the surrounding matrix is not readily discernible.

**Figure 5:**
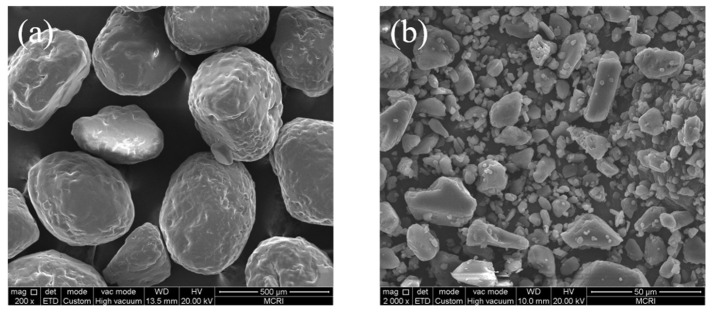
Microscopic shape of (**a**) AP particles and (**b**) HMX particles.

The original microscopic shapes of AP and HMX are illustrated in Figure 5. Comparison of the two revealed that the shape of AP is close to spherical, while the shape of HMX is irregular. This laid the foundation for the subsequent identification of the two different components of the propellant in CT scanning.

### 3.3. Micro-CT Scanning Results

**Figure 6:**
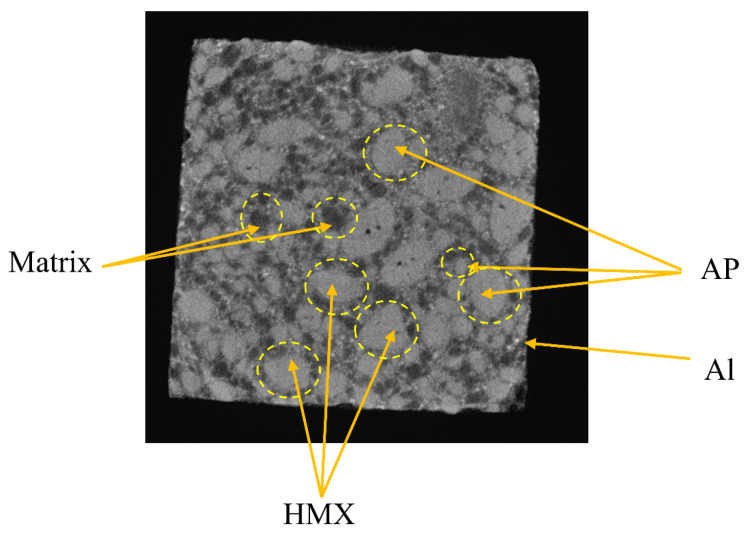
CT image.

CT imaging mainly captures variations in density inside the sample, resulting in distinct grayscale values corresponding to different densities. Figure 6 depicts the unprocessed CT scan picture, enabling differentiation between the particles and matrix. The CT picture depicts the Al particles that possess smaller dimensions and greater densities than compact areas exhibiting elevated luminosity. The slight changes in density and particle size between AP and HMX pose challenges in reliably distinguishing between them. Nevertheless, when observed under SEM, the HMX has an uneven form, but the AP particles seem to be spherical or elliptical. Hence, the AP and HMX particles can be roughly identified in CT images. In the absence of any inherent propellant defects, the matrix density is seen to be the lowest, resulting in a correspondingly lower grey value in the CT picture. As a result, the black region in the image may be identified as the matrix. The identification of Al particles is a challenge due to their significantly smaller size compared to AP and HMX particles. The substantial discrepancy in size hinders the convergence of finite element calculations. Consequently, Al particles are not investigated in isolation, but rather are regarded as part of the matrix.

## 4. Numerical Simulation of Quasi-Static Tensile

In order to carry out numerical simulation of the quasi-static tensile of the propellant, it is essential to establish a microstructural model and mesoscopic finite element model. Notably, the construction of microstructural model in this paper was different from the previous random particle parametric modeling method; instead, it is based on image recognition technology and contour reconstruction, used to construct the microstructure model.

### 4.1. Microstructural Model

#### 4.1.1. Image Recognition Processing

**Figure 7:**
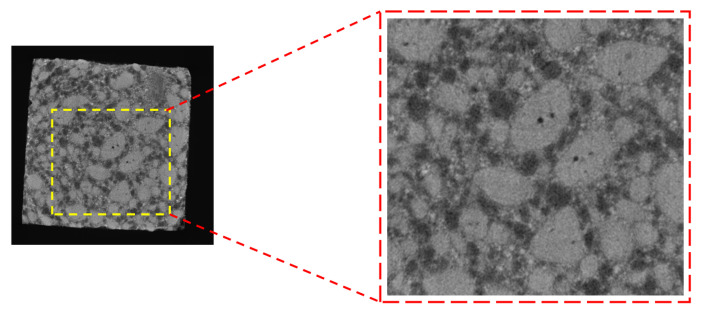
RVE original image.

The initial CT scanning of an image yields a large size, which results in a significant computational burden if employed as a representative volume element (RVE). To mitigate this issue, a suitable RVE size of 800 μm × 800 μm is extracted from the original CT image to serve as the mesoscopic structure of the propellant, as depicted in Figure 7. The captured RVE must contains sufficient meso-structural information to accurately represent the material’s structural heterogeneity on the mesoscopic scale. Additionally, the small size of the RVE allows it to be treated as a mass on the macroscopic scale, ensuring that the material’s approximate homogeneity is preserved.

**Figure 8:**
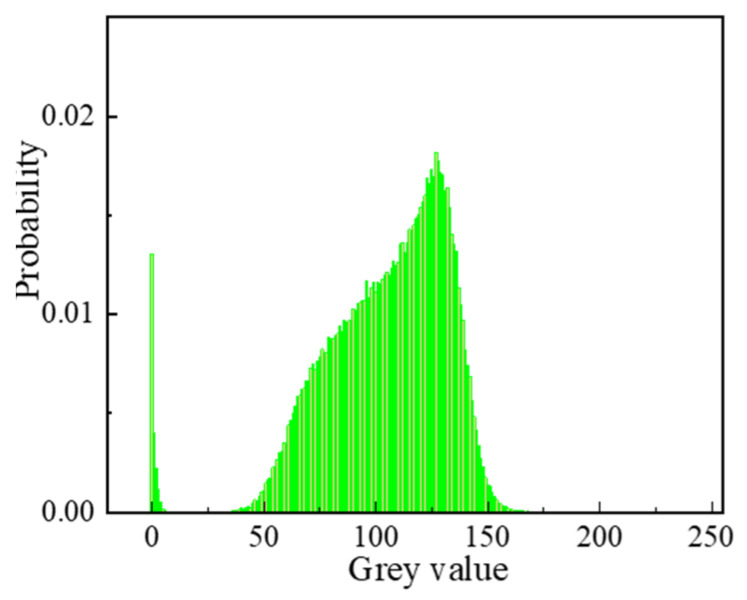
Distribution of grey values in the original image.

**Figure 9:**
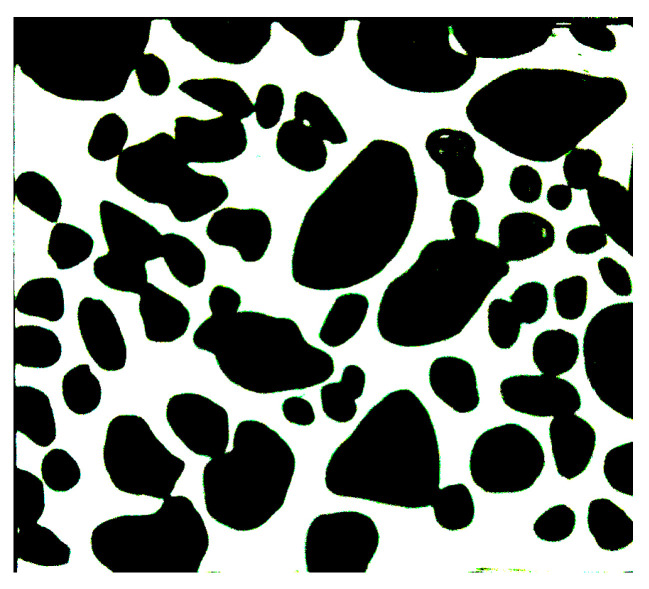
Results of adaptive threshold segmentation.

The original image consists of a matrix whereby each point in the matrix is assigned distinct RGB values, resulting in a variety of colors being shown. To facilitate the subsequent processing, the original image undergoes a procedure known as binarization. The primary objective of this image binarization is to preserve the region of interest containing the relevant features inside the image [39]. Furthermore, the process of threshold segmentation plays an essential part in obtaining an optimal binary image. The statistical analysis reveals that the distribution of grey values in the original image is nonuniform, as shown in Figure 8. The utilization of global threshold segmentation may result in an inappropriate segmentation outcome. Therefore, the present research employs the adaptive threshold (also known as local threshold) segmentation method to perform image thresholding, employing MATLAB 2018b software. The determination of the size of the segmented object in adaptive threshold segmentation processing is influenced by the size moment of the smoothing operator. If the filter size is insufficiently large, the local threshold may not adequately match the required criteria. In general, it is necessary for the width of the smoothing operator to exceed the width of the recognized object. Moreover, a bigger window size tends to yield a more effective smoothed output that may serve as a reliable reference for each individual pixel. Given an input image *I* with a height *H* and width *W*, the adaptive threshold segmentation technique may be executed in the following stages [40]. (1) The picture is subjected to a smoothing operation, applying the median smoothing method and resulting in the smoothed image denoted as $f_{smooth}(I)$. (2) To obtain the adaptive thresholding matrix, $Thresh=(1-ration)*f_{smooth}(I)$, where ration is equal to 0.15. (3) The process of segmentation is executed by employing local threshold segmentation rules. The results of the process of segmentation are depicted in Figure 9. It is evident from this observation that the particles largely retain their initial shape, with localized instances of particle stacking.

#### 4.1.2. Microstructural Modeling

To construct a microstructural model suitable for importing into the Abaqus software for computational analysis, it is imperative to accurately identify and convert the contour lines of the RVE into vector format following the process of binarization. The particle edge contour derived from image recognition has a coarse nature, making it challenging to achieve convergence during finite element calculations conducted on this foundation. Therefore, it is important to make an approximation of the contour to obtain a more optimal convergent form. The circularization of particle edges was achieved by adopting the fitting approach suggested by Wang et al. [41], which relies on the automated extraction of segmented points. The contour fitting algorithm involves the automated extraction of segmented points, followed by the determination of least-squares fitting equations with unknown coefficients for these segmented point intervals. The algorithm subsequently solves the least-squares error and objective function, incorporating constraints through the Lagrange multiplier method. This process ultimately yields a complete, continuous, and smooth contour. The Lagrange multiplier method transforms an optimization problem with constraints into an unconstrained problem, and satisfies the constraints of the original problem by introducing Lagrange multipliers. Moreover, this method has the advantages of simple algorithm and easy implementation, and performs well in convex optimization problems [42,43]. To realize the accurate fitting of the particle contour, the discrete points comprising the contour are fitted and segmented by setting appropriate error and threshold values. The method to determine the segmentation points is as follows.

A polynomial least-squares curve is employed to approximate the discrete points {(*x*_1_, *y*_1_), (*x*_2_, *y*_2_),…, (*x*_n_, *y*_n_)} on the contour. The fitting process is carried out in the positive direction of the *X*-axis using *q* data points. The threshold for the fitting error, denoted as *e*(*u*), is determined based on the specified error criterion. The function *e*(*u*) is dependent on the number of fitted points *u*, which is expressed as follows.

(1) $$e\left(u\right)=wu$$

where *w* is the error unit constant that is determined based on the error requirement.

The first segment point is determined by setting *k* = 1. The first polynomial least-squares fitting for the *k*th segment curve is performed in a left-to-right manner with the discrete points on the contour. Specifically, the fitting equation is derived by considering the first data point to the *q*th data point on the contour line, and the equation is as follows:

(2) $$F_{k1}\left(x\right)=\sum_{i=1}^{R+1}a_{1i}^{\left(1\right)}x^{R-i},x⩽x_{q}$$

where *R* is the highest polynomial order.

The initial fitting error of the *k*th curve *E*_k1_ is calculated using the following formula:

(3) $$E_{k1}=\sum_{i=1}^{q}\left|F_{11}\left(x_{i}\right)-y_{i}\right|$$

The amount of the fitting error *E_k_*_1_ is compared with the threshold *e*(*q*). If the fitting error satisfies the following criteria:

(4) $$E_{k1}⩽e\left(q\right)$$

then a secondary fitting process is conducted for the *k*th curve. Particularly, a polynomial least-squares fitting method is employed for the *k*th curve, commencing from the initial data point, and extending to the 2*q*th data point. The equation for fitting and the error associated with the fitting can be expressed as follows.

(5) $$F_{k2}\left(x\right)=\sum_{i=1}^{R+1}a_{1i}^{\left(2\right)}x^{R-i},x⩽x_{2q}$$

(6) $$E_{k2}=\sum_{i=1}^{2q}\left|F_{12}\left(x_{i}\right)-y_{i}\right|$$

Similarly, a determination is made as to whether the fitting error *E_k_*_2_ satisfies the threshold. If the condition *E_k_*_2_ ≤ *e*(2*q*) is fulfilled, then proceed to the subsequent step with the approach described above. The *k*th curve fitting process is considered complete when the fitting error *E_k_*_(*mk*+1)_ > *e* [(*m_k_* + 1) *q*] for *m_k_* + 1 times.

At each segmentation point, a collection of *p* data points is extracted in both the negative and positive directions of the X-axis, with the segmentation point serving as the center. These segmentation points together comprise the data set *X*_1_, *X*_2,_…, *X_K_*.

(7) $$\begin{array}{c} X_{1}=\left\{\left(x_{1}^{\left(1\right)},y_{1}^{\left(1\right)}\right),\left(x_{2}^{\left(1\right)},y_{2}^{\left(1\right)}\right),\cdots ,\left(x_{2p+1}^{\left(1\right)},y_{2p+1}^{\left(1\right)}\right)\right\}\\ X_{2}=\left\{\left(x_{1}^{\left(2\right)},y_{1}^{\left(2\right)}\right),\left(x_{2}^{\left(2\right)},y_{2}^{\left(2\right)}\right),\cdots ,\left(x_{2p+1}^{\left(2\right)},y_{2p+1}^{\left(2\right)}\right)\right\}\\ \vdots \\ X_{K}=\left\{\left(x_{1}^{\left(K\right)},y_{1}^{\left(K\right)}\right),\left(x_{2}^{\left(K\right)},y_{2}^{\left(K\right)}\right),\cdots ,\left(x_{2p+1}^{\left(K\right)},y_{2p+1}^{\left(K\right)}\right)\right\} \end{array}$$

where *K* is the total number of segment points.

Polynomial least-squares curve fitting has been performed on the data points for each major segmentation interval in the process of determining the segmentation points. The fitting equation is denoted as

(8) $$\left\{\begin{array}{c} F_{1}\left(x\right)=F_{m_{1}}\left(x\right)=\sum_{i=1}^{R+1}a_{1i}^{\left(m_{1}\right)}x^{R-i},x⩽x_{1}^{\left(1\right)}\\ F_{2}\left(x\right)=F_{m_{2}}\left(x\right)=\sum_{i=1}^{R+1}a_{2i}^{\left(m_{2}\right)}x^{R-i},x_{2p+1}^{\left(1\right)}⩽x⩽x_{1}^{\left(2\right)}\\ \vdots \\ F_{K+1}\left(x\right)=F_{m_{K+1}}\left(x\right)=\sum_{i=1}^{R+1}a_{\left(K+1\right)i}^{\left(m_{K+1}\right)}x^{R-i},x_{2p+1}^{\left(K\right)}⩽x \end{array}\right.$$

where *m*_j_ is the number of fits required to obtain the curve. The curve fitting equation is *F_mj_*(*x*) for the *j*th segmented interval, *j* = 1, 2,…, *K* + 1.

Let the curve fitting equation expression for each segmented point interval be

(9) $$\left\{\begin{array}{c} f_{1}\left(x\right)=\sum_{i=1}^{r+1}a_{1i}x^{r-i},x_{1}^{\left(1\right)}⩽x⩽x_{2p+1}^{\left(1\right)}\\ f_{2}\left(x\right)=\sum_{i=1}^{r+1}a_{2i}x^{r-i},x_{1}^{\left(2\right)}⩽x⩽x_{2p+1}^{\left(2\right)}\\ \vdots \\ f_{K}\left(x\right)=\sum_{i=1}^{r+1}a_{Ki}x^{r-i},x_{1}^{\left(K\right)}⩽x⩽x_{2p+1}^{\left(K\right)} \end{array}\right.$$

where *r* is the highest order of the polynomial fitting curve for the segmented point interval, and {*a_ki_*}*_k_* _= 1, 2,…, *K*; *i* = 1, 2,…, *r*+1_ are the unknown coefficients to be determined.

The Lagrange multiplier method is employed to address the problem of minimizing the least-squares error while considering restrictions. This approach ensures that the segmented point intervals and the major segmented intervals are connected in a smooth manner, resulting in the formation of an entire contour curve. The least-squares objective function may be derived based on the fitted equations for the segmented point intervals.

(10) $$\underset{k=1,2,\cdots ,K}{min}Z_{k}=\sum_{i=1}^{2p+1}{\left[f_{k}\left(x_{i}^{\left(k\right)}\right)-y_{i}\right]}^{2}$$

The following are the constraint conditions for the continuity and smoothing of segmented curves:

(11) $$\left\{\begin{array}{c} f_{k}\left(x_{1}^{\left(k\right)}\right)=F_{k}\left(x_{1}^{\left(k\right)}\right)\\ f_{k}\left(x_{2p+1}^{\left(k\right)}\right)=F_{k+1}\left(x_{2p+1}^{\left(k\right)}\right)\\ f_{k}^{\prime }\left(x_{1}^{\left(k\right)}\right)=F_{k}^{\prime }\left(x_{1}^{\left(k\right)}\right)\\ f_{k}^{\prime }\left(x_{2p+1}^{\left(k\right)}\right)=F_{k+1}^{\prime }\left(x_{2p+1}^{\left(k\right)}\right) \end{array}\;\;\;\;k=1,2,\cdots ,K\right.$$

According to the constraints, let

(12) $$\left\{\begin{array}{c} H_{k1}=f_{k}\left(x_{1}^{\left(k\right)}\right)-F_{k}\left(x_{1}^{\left(k\right)}\right)\\ H_{k2}=f_{k}\left(x_{2p+1}^{\left(k\right)}\right)-F_{k+1}\left(x_{2p+1}^{\left(k\right)}\right)\\ H_{k4}=f_{k}^{\prime }\left(x_{1}^{\left(k\right)}\right)-F_{k}^{\prime }\left(x_{1}^{\left(k\right)}\right)\\ H_{k4}=f_{k}^{\prime }\left(x_{2p+1}^{\left(k\right)}\right)-F_{k+1}^{\prime }\left(x_{2p+1}^{\left(k\right)}\right) \end{array}\;\;\;\;k=1,2,\cdots ,K\right.$$

The function is then solved by using Lagrange multipliers to obtain the unknown coefficients of the fitted function for the interval of each segmentation point. The Lagrange function, which is established via the introduction of Lagrange multipliers, can be expressed as follows.

(13) $$L_{k}\left(a_{k1},a_{k2},\cdots ,a_{kr},\lambda_{k1},\lambda_{k2},\lambda_{k3},\lambda_{k4}\right)=Z_{k}+\lambda_{k1}H_{k1}+\lambda_{k2}H_{k2}+\lambda_{k3}H_{k3}+\lambda_{k4}H_{k4}$$

**Figure 10:**
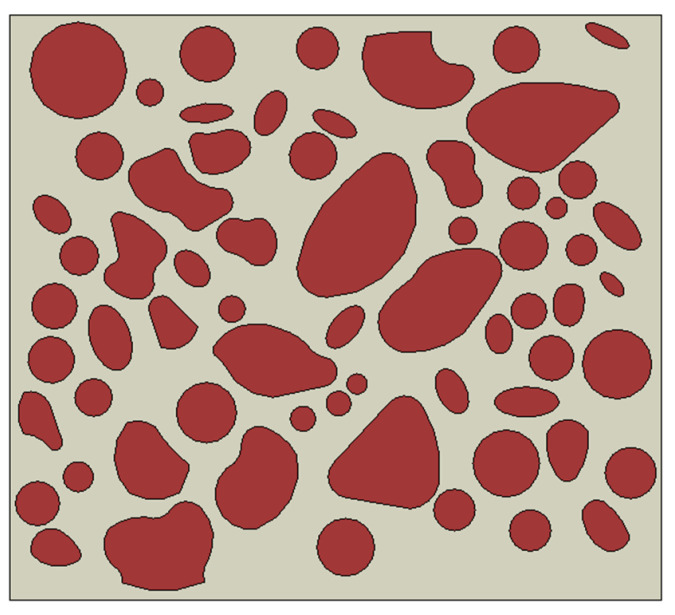
The mesoscopic structured model after the approximate treatment.

The partial derivatives of the unknown coefficients *a_ki_*, *i* = 1, 2,…, *r* + 1 and $\lambda_{ki}$, *i* = 1, 2, 3, 4 in the Lagrange function *L_k_* can be obtained by considering the essential conditions for the extremum of the multivariate function, and the expression is as follows.

(14) $$\left\{\begin{array}{c} \frac{\partial L_{k}}{\partial a_{ki}}=\frac{\partial Z_{k}}{\partial a_{ki}}+\lambda_{k1}\frac{\partial H_{k1}}{\partial a_{ki}}+\lambda_{k2}\frac{\partial H_{k2}}{\partial a_{ki}}+\lambda_{k3}\frac{\partial H_{k3}}{\partial a_{ki}}+\lambda_{k4}\frac{\partial H_{k4}}{\partial a_{ki}}\\ \frac{\partial L_{k}}{\partial \lambda_{kj}}=H_{kj} \end{array}\right.$$

where *k* = 1, 2,…, *K*. By setting Equation (14) to equal zero, the system of equations can be solved to yield the unknown coefficients *a_ki_*, *i* = 1, 2,…, *r* + 1. The coefficient is then inserted into Equation (9) to obtain the equation of the curve for each segmented point interval. This, in conjunction with Equation (8), results in a complete and continuous contour of the fitted equation. Finally, a filling model with more regular particle shapes was obtained, as shown in Figure 10.

### 4.2. Mesoscopic Finite Element Model

#### 4.2.1. Meshing and Periodic Boundary Conditions

The particles and matrix exhibit different material characteristics, necessitating the independent definition of element types at the meshing stage in finite element modelling. Furthermore, due to the presence of irregular particles in the model, the process of mesh delineation becomes more formidable. AP and HMX are crystals, and the binder matrix is a polymer in composite solid propellants. Furthermore, AP and HMX are intercalated in the matrix, which is equivalent to the reinforcing phase of particle-reinforced polymer composites. Comparatively, AP and HMX are rigid bodies. Therefore, the elastic modulus of AP and HMX particles is significantly greater than that of the matrix. Consequently, it can be inferred that the particles will not induce substantial deformation under load. As a result, the appropriate choice for the element type is the plane strain element CPE3. Based on the assumptions of plane strain and incompressibility, the volume of the matrix is kept constant under load; thus, the element type of the matrix is chosen as plane strain hybrid element CPE3H.

**Figure 11:**
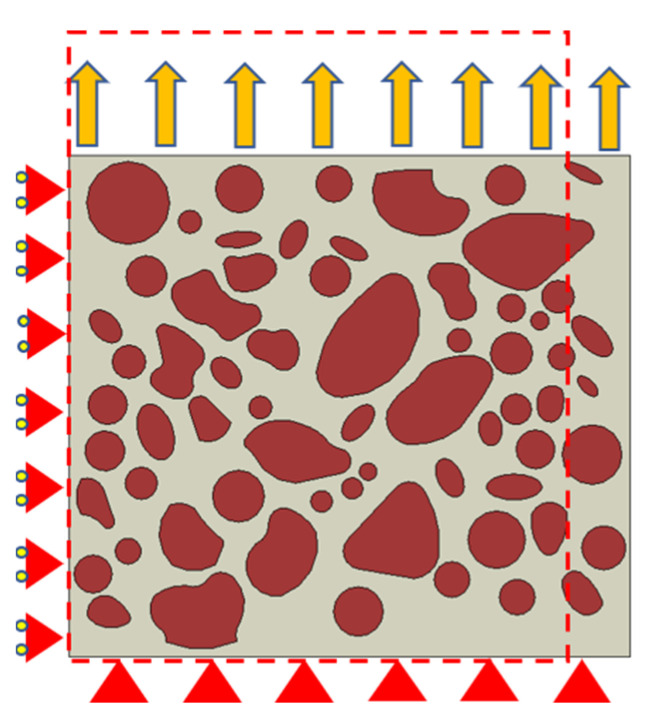
Schematic illustration of boundary conditions.

Based on the periodic hypothesis, the internal stress/strain fields of the propellant exhibit continuity and periodicity under the external loading. Hence, it is essential to implement periodic boundary conditions on the RVE in finite element analysis. This is carried out to ensure the fulfilment of the assumptions of continuity and homogeneity at the macroscopic level, and to achieve a rational distribution of stress and strain. The schematic illustration of symmetric boundary conditions is shown in Figure 11. The periodic boundary conditions can be expressed as follows [44]:

(15) $$\left\{\begin{array}{c} d\left(x,0\right)-d_{1}=d\left(x,L\right)\\ d\left(0,y\right)-d_{2}=d\left(L,y\right) \end{array}\right.$$

where *L* is the RVE size, and *d*_1_ and *d*_2_ are the border displacements.

#### 4.2.2. Constitutive Model and Parameters of Materials

The binder matrix of a composite solid propellant possesses superelasticity and viscoelasticity at ambient temperature, according to our references [45,46]. Referring to the simulation approach in reference [45], only the superelasticity of the matrix was observed in this research to increase convergence, while its viscoelasticity was ignored, and the Mooney–Rivlin superelastic model was employed as the matrix’s constitutive model. The Mooney–Rivlin model is renowned for its inherent simplicity, with its constitutive model being represented by the strain energy function *W.* The expression of elastic strain potential energy is as follows [47]:

(16) $$W=C_{10}\left(I_{1}-3\right)+C_{01}\left(I_{2}-3\right)+\frac{1}{D_{1}}{\left(J-1\right)}^{2}$$

where *C*_10_ and *C*_01_ are the hyperelastic parameters of the matrix, which need to be fitted using experimental data. *D*_1_ is the incompressible parameter of the material. *J* is the elastic volume ratio. If the material is assumed to be incompressible, then *J* = 1, and the third term is 0. The parameters of the constitutive model were obtained by fitting the experimental data based on the reference [47], as shown in Table 2.

The composite solid propellant belongs to a representative example of particle-filled composites with a high solid content. The filled particles possess a much greater modulus and strength compared to the binder matrix. The reference [48] demonstrates that under quasi-static stress conditions, the interior particles of the propellant remain undamaged and may be considered elastic materials. The pertinent characteristics of AP and HMX were acquired based on the references cited as [49,50], and are shown in Table 3.

#### 4.2.3. Cohesive Zone Model

The presence of distinct interfaces between the particles and matrix of the propellant is readily apparent, and interface damage stands out as a primary factor contributing to propellant degradation and ultimate destruction. The cohesive zone model is commonly employed to characterize the mechanical response of the interface during numerical simulations with the microstructural model, with the aim of simulating the process of interface damage. The bilinear model and the exponential model are often employed as cohesive models for simulating the mechanical behavior of microstructural models. In contrast with the exponential model, the bilinear model offers the benefits of simplicity and convenience in obtaining the associated parameters. Hence, the mechanical behavior of the contact is described in this study using the bilinear cohesive model.

**Figure 12:**
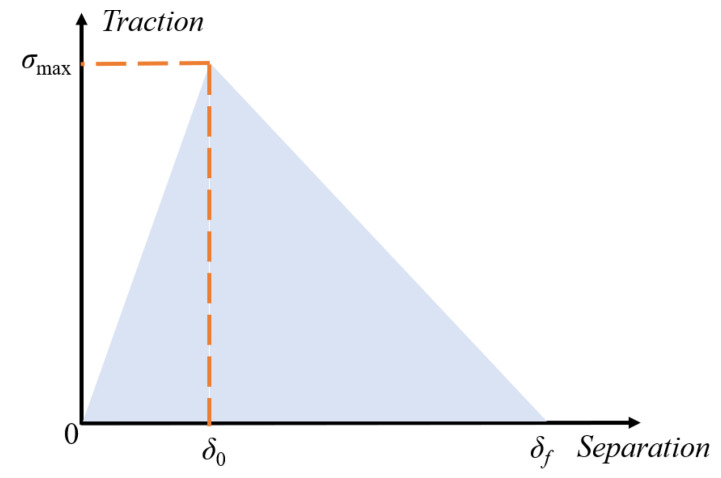
Schematic diagram of a bilinear cohesive zone model.

The typical bilinear cohesive model is formulated based on fracture mechanics, which primarily focuses on characterizing the initiation and evolution of damage by defining the traction displacement law, as shown in Figure 12 [49]. The traction displacement law is described as follows [51]:

(17) $$T=\left(1-D\right)\frac{\delta }{\delta_{0}}\sigma_{max}$$

where *T* is the traction force. *δ* is the interfacial separation displacement. *σ_max_* is the interfacial bond strength. *δ*_0_ is the interfacial damage onset displacement. *D* is the damage variable, defined by the following equation:

(18) $$D=\left\{\begin{array}{l} 0\;\;\;\;\;\;\;\;\;\;\;\;\;\;\;\;\;\;\;\;\;\;\;\;\left(\delta \ll \delta_{0}\right)\\ \frac{\delta_{f}\left(\delta -\delta_{0}\right)}{\delta \left(\delta_{f}-\delta_{0}\right)}\;\;\;\;\;\;\;\;\;\;\;\;\left(\delta >\delta_{0}\right) \end{array}\right.$$

where *δ_f_* is the failure displacement at the interface.

**Figure 13:**
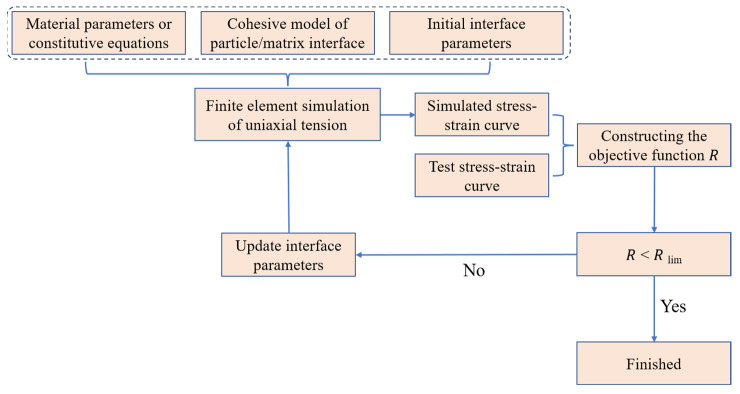
Inversion flow of the interface model’s parameters.

It can be revealed from the above analysis that the basic parameters of a typical bilinear model mainly consist of interface stiffness *K*, interface strength *σ*, and failure displacement *δ*. Obtaining interfacial model parameters with experimental methods is challenging due to the constraints imposed by experimental methodologies and theories. Numerous investigators have conducted similar studies to ascertain the values of model parameters by employing methods from the literature and empirical estimation. However, the outcomes frequently exhibit inaccuracies. On this basis, this work utilizes the parameter inversion approach based on the work of Hooke–Jeeves to determine the ultimate interfacial parameters. The process of inversion is shown in Figure 13. The objective function utilized for assessing the coincidence degree between the experimental curve and simulation curve is as follows [2]:

(19) $$R=\frac{1}{n}\sum_{i=1}^{n}{\left[\sigma_{\varepsilon_{i},\;\;sim}\left(K,\sigma_{max},\delta \right)-\sigma_{\varepsilon_{i},\;\;exp}\right]}^{2}$$

where $\sigma_{\varepsilon_{i},\;sim}$ and $\sigma_{\varepsilon_{i},\;exp}$ represent the stress obtained from numerical simulation and the stress obtained from testing at a certain strain, respectively.

Prior to the occurrence of parameter inversion, it is necessary to provide a predetermined set of initial values for the model. Additionally, the permissible tolerance *R_lim_* is gradually approximated through several iterations. If the value of objective function *R* is less than the specified tolerance *R_lim_*, the parameter values can be determined as the final parameters of the interface model.

## 5. Numerical Simulation Results and Discussion

### 5.1. Stress—Strain Curves

**Figure 14:**
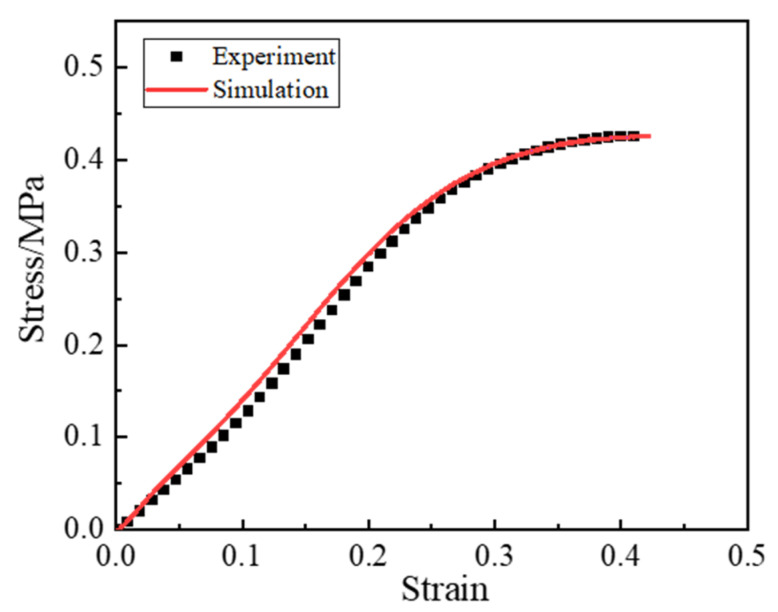
The stress–strain curves were obtained via simulation at 1 mm/min (0.000119 s^−1^).

Based on the microstructural model established in Section 4, the quasi-static tensile properties of the propellant were simulated by applying an equal displacement load with a rate of 1 mm/min. The stress–strain curves of the composite solid propellant were derived through simulation, and are shown in Figure 14. According to Figure 14, the macroscopic mechanical response curves of the solid propellant obtained from the simulation exhibit a notable agreement with the corresponding experimental results. Comparing the values of simulation and experiment, when the strain is 0.14, the corresponding stress error is largest, its value is 0.018 MPa, and the overall average relative error is 5.4%.

The stress–strain curve of the propellant subjected to uniaxial tensile force, which may be approximately categorized into three distinct stages. (1) The linear elastic stage, in which stress increases linearly with strain and no damage occurs at the propellant interface. (2) In the dewetting stage, the stress tends to decrease as the strain increases, material stiffness seems to decrease, and dewetting damage emerges in the larger particles or dense sections of particles. The overall stress continues to rise. (3) In the stress reduction stage, as strain increases, a “stress drop zone” appears, and the dewetting displacement of the interface of large particles increases. The effect of the load on the particles decreases, the polar parts of the particles are gradually exposed themselves, and the matrix’s bearing capacity increases.

### 5.2. Damage Evolution Process

**Figure 15:**
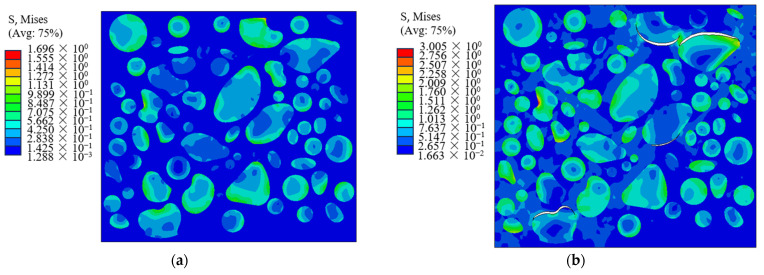

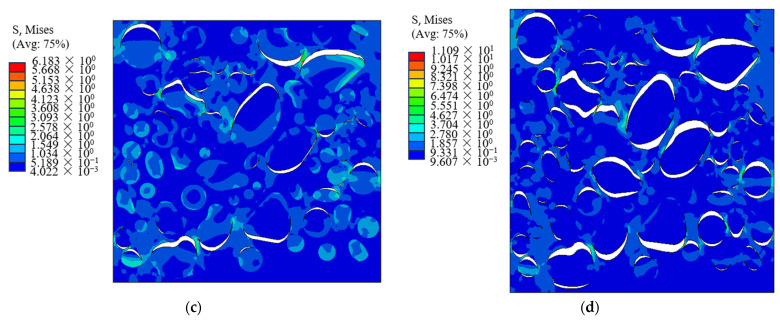
Mesoscopic morphology corresponding to different strains. (**a**) Strain of 2%; (**b**) Strain of 5%; (**c**) Strain of 20%; (**d**) Strain of 40%.

The microstructural model is used to simulate the damage process of the propellant under uniaxial tensile load, and the microstructural morphology corresponding to different strains at a 1 mm/min tensile rate is shown in Figure 15. It is evident that during the initial stage of load application, namely when the strain reaches 2%, the primary function of internal particles within the propellant is to bear the load. This may be attributed to the fact that the elastic modulus of the particles is significantly greater than that of the matrix. Once the applied strain reaches 5%, the occurrence of dewetting becomes evident in the vicinity of the bigger particles, leading to the formation of microcracks. The phenomena can be attributed to the comparatively weak contact between the particles and the matrix, as well as the lesser deformation of the particles compared to that of the matrix during the tensile process. At a strain level of 20%, there is an observed increase in the dewetting phenomenon occurring between the particles and the matrix. This is because when the strain reaches 20%, many different shapes of larger particles along the interface in the loading direction are the first microcracks or micropores to appear. This indicates that the large particles are prone to dewetting, and the dewetting location first appears in the larger particles and particles’ aggregation area. The aggregation area of larger particles forms stress bridging due to the interaction between particles, resulting in a stress concentration phenomenon, which leads to a more serious dewetting phenomenon. Additionally, the presence of microcracks becomes evident in regions characterized by a high density of small particles. The reason for the continued advancement of dewetting is that the local strain in the vicinity of bigger particles is larger, particularly in the regions with dense particles. Consequently, the local stress interaction between these particles is intensified. Upon reaching the strain level of 40%, the fractures and pores resulting from particle dewetting exhibit a tendency to merge, ultimately leading to complete failure. Once the interface has undergone full dewetting, the particles cease to carry any load, leading to the occurrence of stress concentration at the crack tip. The continued application of the load will result in the convergence of the fractures, ultimately resulting in the matrix’s structural failure. In conclusion, it is noticeable that particle dewetting serves as the main trigger for the destruction of the propellant, a phenomenon that aligns well with the experimental findings presented in Section 2.

## 6. Conclusions

The results of this study led to the following conclusions.

1. The results of uniaxial tensile tests under three different strain rates (0.000119 s^−1^, 0.000476 s^−1^ and 0.00119 s^−1^) at ambient temperature revealed that the mechanical properties of the HTPPB propellant have a significant rate dependence. The greater the strain rate, the greater the maximum tensile strength of the propellant. When the strain rate is 0.00119 s^−1^, the maximum tensile strength is 0.48 MPa, and when the strain rate is 0.000119 s^−1^, the minimum tensile strength is 0.37 MPa. In addition, the results obtained from the SEM experiments indicated that the destruction of the propellant at low strain rates primarily occurs due to interfacial damage between larger particles (AP and HMX) and the matrix, referred to as “dewetting”. However, the “dewetting” phenomenon is not clearly observed in the case of the smaller particles (Al).
2. The interior mesoscopic structure of the propellant may be more accurately characterized by employing a microstructural model that is generated using micro-CT scans and image-processing technologies. Moreover, it is observed that the change trend remains consistent. This finding suggested that the established microstructural model exhibits a high level of reliability and offers a novel approach for simulating propellants that contain particles in future studies.
3. The mesoscopic finite element model based on the hyperelastic constitutive model of the matrix and the bilinear cohesive model can better describe the mechanical response of the propellant. The model enables the study of a mesoscopic damage evolution process, thus indirectly elucidating the damage mechanism.
4. In future work, it is hoped that a three-dimensional microstructural model that can reflect the real mesoscopic structure and a cohesive zone model with temperature dependence and rate dependence can be established as the basis for numerical simulations to accurately obtain the correlation between the evolution in the propellant microstructure and the macroscopic mechanical response. This will help to guide the design of composite solid propellant formulations and the assessment of the structural integrity of solid rocket motor charges.

## Figures and Tables

**Table 1:** Proportion of composite solid propellant components.

Components	Density/(g/cm^3^)	Mass Fraction	Particle Size
AP	1.95	48%	50–300 μm
HMX	1.96	22%	50–120 μm
Al	2.7	20%	5–18 μm
HTPB	0.9	9%	-
Others	0.9	1%	-

**Table 2:** Parameters of the Mooney–Rivlin model.

$C_{10}$	$C_{01}$	*R* ^2^
0.00677	0.0293	0.965

**Table 3:** Mechanical parameters of particles.

Particle	Elastic Modulus/GPa	Poisson’s Ratio
AP	32.45	0.14
HMX	17.48	0.25

## Data Availability

Data will be made available on request.

## References

[1] Wang J.X., Qiang H.F., Wang Z.J. (2020). Research progress on mesomechanics of composite solid propellants. J. Solid Rocket. Technol..

[2] Han L., Xu J.S., Zhou C.S. (2016). Inverse Identification of the Rate-dependent Micro Interface Parameters of HTPB/IPDI Composite Propellant. Chin. J. Energetic Mater..

[3] Han L., Chen X., Xu J.S., Zhou C.S., Zhao L. (2017). Relaxation behavior of composite propellant based on meso-mechanical model. J. Solid Rocket. Technol..

[4] Zhao J.L., Qiang H.F. (2011). Interface damage bi-scale simulation of composite solid propellant based on cohesive work. J. Solid Rocket. Technol..

[5] Hou Y.F., Xu J.S., Zhou C.S., Chen X. (2021). Microstructural simulations of debonding, nucleation, and crack propagation in an HMX-MDB propellant. Mater. Des..

[6] Sheng Y.P., Jia H.C., Lv H.L., Chen H.X., Zhao X.R., Wang R.Z., Meng J.D. (2020). Study on Mesoscopic Mechanics of Recycled Asphalt Mixture in the Indirect Tensile Test. Math. Probl. Eng..

[7] Ostoja-Starzewski M., Kale S., Karimi P., Malyarenko A., Raghavan B., Ranganathan S.I., Zhang J. (2016). Scaling to RVE in Random Media. Adv. Appl. Mech..

[8] Zhang X., Zhang S., Jia Y.F., Liu C.Y., Gao X.G., Wang F., Song Y.D. (2023). A parameterized and automated modelling method for 3D orthogonal woven composite RVEs considering yarn geometry variations. Compos. Struct..

[9] Henrich M., Fehlemann N., Bexter F., Neite M., Kong L.H., Shen F.H., Konemann M., Dolz M., Munstermann S. (2023). DRAGen—A deep learning supported RVE generator framework for complex microstructure models. Heliyon.

[10] Sun Q.P., Jian M.K. (2022). Computational Elastic Analysis of AA7075-O using 3D-Microstructrure—Based-RVE with Really-distributed Particles. Int. J. Mech. Sci..

[11] Shokrieh M.M., Moshrefzadeh-Sani H. (2016). An optimized representative volume element to predict the stiffness of aligned short fiber composites. J. Compos. Mater..

[12] Firooz S., Saeb S., Chatzigeorgiou G., Meraghni F., Steinmann P., Javili A. (2019). Systematic study of homogenization and the utility of circular simplified representative volume element. Math. Mech. Solids.

[13] Meng L., Tabiei A. (2021). An irreversible bilinear cohesive law considering the effects of strain rate and plastic strain and enabling reciprocating load. Eng. Fract. Mech..

[14] Zhang W.L., Tabiei A. (2018). Improvement of an Exponential Cohesive Zone Model for Fatigue Analysis. J. Fail. Anal. Prev..

[15] Ghabezi P., Farahani M. (2017). Effects of Nanoparticles on Nanocomposites Mode I and II Fracture: A Critical Review. Rev. Adhes. Adhes..

[16] Ghabezi P., Farahani M. (2018). Trapezoidal traction-separation laws in mode II fracture in nano-composite and nano-adhesive joints. J. Reinf. Plast. Compos..

[17] Ghabezi P., Farahani M. (2019). A cohesive model with a multi-stage softening behavior to predict fracture in nano composite joints. Eng. Fract. Mech..

[18] Musto M., Alfano G. (2015). A fractional rate-dependent cohesive-zone model. Int. J. Numer. Methods Eng..

[19] Zhou X., Hao H. (2008). Mesoscale modelling of concrete tensile failure mechanism at high strain rates. Comput. Struct..

[20] Jin L., Yu W., Du X., Zhang S., Li D. (2019). Meso-scale modelling of the size effect on dynamic compressive failure of concrete under different strain rates. Int. J. Impact Eng..

[21] Jin L., Yu W., Du X., Yang W. (2019). Mesoscopic numerical simulation of dynamic size effect on the splitting-tensile strength of concrete. Eng. Fract. Mech..

[22] Xu W., Chen H. (2012). Mesostructural characterization of particulate composites via a contact detection algorithm of ellipsoidal particles. Powder Technol..

[23] Zhou R., Song Z., Lu Y. (2017). 3D mesoscale finite element modelling of concrete. Comput. Struct..

[24] Ma H., Yue C., Yu H., Mei Q., Chen L., Zhang J., Zhang Y., Jiang X. (2020). Experimental study and numerical simulation of impact compression mechanical properties of high strength coral aggregate seawater concrete. Int. J. Impact Eng..

[25] Zhang Z., Song X., Liu Y., Wu D., Song C. (2017). Three-dimensional mesoscale modelling of concrete composites by using random walking algorithm. Compos. Sci. Technol..

[26] Hafner S., Eckarat S., Luther T. (2006). Mesoscale modelling of concrete: Geometry and numerics. Comput. Struct..

[27] Li S., Li Q. (2015). Method of meshing ITZ structure in 3D meso-level finite element analysis for concrete. Finite Elem. Anal. Des..

[28] Zhu Z., Chen H., Xu W., Liu L. (2014). Parking simulation of three-dimensional multi-sized star-shaped particles. Model. Simul. Mater. Sci. Eng..

[29] Liu Z.Q., Li G.C., Xing Y.G., Dong K.H., Chen X.M. (2011). Numerical simulation and SEM study on the microstructural damage of composite solid propellants. J. Propuls. Technol..

[30] Han L. (2016). Research on the Mesoscopic Damage Mechanism and Nonlinear Viscoelastic Constitutive Model of Composite Propellant. Ph.D. Thesis.

[31] Widom B. (1965). Random sequential addition of hard spheres to a volume. J. Chem. Phys..

[32] Rintoul M.D., Torquato S. (1997). Reconstruction of the structure of dispersions. J. Colloid Interface Sci..

[33] Jackson T., Stafford D.S. (2010). Random Packs and Their Use in Grain-Scale Modeling, with Applications to Energetic Materials. Int. J. Multiscale Comput. Eng..

[34] Stafford D.S., Jackson T.L. (2010). Using level sets for creating virtual random packs of non-spherical convex shapes. J. Comput. Phys..

[35] Garboczi E.J., Bullard J.W. (2013). Contact function, uniform-thickness shell volume, and convexity measure for 3D star-shaped random particles. Powder Technol..

[36] Zhao B.D., Wang J.F. (2016). 3D quantitative shape analysison form, roundness, and compactness with µCT. Powder Technol..

[37] (2005). The People’s Republic of China GJB 770B—2005: Test Method of Propellant.

[38] Wu C., Lu Y., Jiang M., Hu S., Yang H., Fu X., Li H. (2023). Study on Mechanical Properties and Failure Mechanisms of Highly Filled Hydroxy-Terminated Polybutadiene Propellant under Different Tensile Loading Conditions. Polymers.

[39] Tan R.H., Hu X.P., Peng X.Q. (2022). A line fitting algorithm based on contour edge. Chin. J. Sens. Actuators.

[40] Zhao C.H., Wang J., Wang Y.L. (2016). Hyperspectral anomaly detection based on background suppression and adaptive threshold segmentation. J. Harbin Eng. Univ..

[41] Wang L., Xin Z.Y., Chen S. (2017). A wheel profile fitting method based on automatically extracting subsection points. J. Guangxi Univ..

[42] Burman E., Hansbo P., Larson M.G. (2023). The Augmented Lagrangian Method as a Framework for Stabilised Methods in Computational Mechanics. Arch. Comput. Methods Eng..

[43] Jafari H., Alipoor A. (2011). A New Method for Calculating General Lagrange Multiplier in the Variational Iteration Method. Numer. Methods Partial Differ. Equ..

[44] Feng T., Xu J.S., Han L., Chen X., Zhou C.S. (2018). Mechanical properties of composite solid propellant with initial defects. J. Aeronaut. Mater..

[45] Zhang Z.G., Hou X., Gao J., Wang L. (2019). A method of generating two-dimensional mesoscopic model for hydrox-yl-terminated polybutadiene propellant with high particle volume fraction. Acta Mater. Compos. Sin..

[46] Maimaitttuersun W., Wu Y.Q., Hou X., Wang N. (2022). Numerical investigations on mesoscopic structure parameters affecting mechanical responses of propellant. Acta Mater. Compos. Sin..

[47] Chang W.J. (2013). Research on Microstructural Damage and Its Numerical Simulation Method for Composite Solid Propellant. Master’s Thesis.

[48] Feng T., Zheng J., Xu J.S., Han L., Kang N. (2018). Mesoscopic structure modeling and numerical simulation of debonding process of composite solid propellants. J. Aerosp. Power.

[49] Wang J.X., Qiang H.F., Wang Z.J. (2020). Parameterized meso-scale modeling of composite solid propellant. J. Rocket. Force Univ. Eng..

[50] Wang X.J., Wu Y.Q., Huang F.L. (2015). Nanoindentation experiments and simulations studies on mechanical responses of energetic crystals. Chin. J. Theor. Appl. Mech..

[51] Wu C., Liu Y., Hu S., Lu Y., Guo C., Li H., Qu H., Fu X., Li H. (2023). Correlation between microstructural evolution and mechanical properties of CMDB propellant during uniaxial tension. Propellants Explos. Pyrotech..

